# Book Review

**Published:** 1930

**Authors:** 


### Reviews of Books

**The Doctor's Job.** By A. Lloyd-Williams, M.B., B.S. Pp. vii., 72. London: Student Christian Movement Press. 1930. Price 2s.—A study of medical life and religious character. The author moves in a world which perplexes students and tries the honesty of practitioners; where instincts and passions, fear and care are components of disease, and the healers of the time are fain to find in Christian sources a cue to conquer pain or inspire hope. What, in this sort, a sympathetic understanding may do for one's neighbour is illustrated in a case of broken health repaired, broken vows and broken fortune. The foreword commends the author's helpfulness and sagacity. In handling authorities, in apologetic, in appeal, through various topics of social import—the deep wells of adolescence and maturity, the future of hospital or race, the weaker vessel and her vindication—a right judgment in all things is preserved. We may dissent, though we gather with the sons of the prophets there is death in the pot if accommodation be allowed to those who refuse to increase and multiply. Hold we, too, that, rejected by efficiency tests, there is still that in wine which cheers God and man. These candid pages of the author's *Horae subsecivae* enforce the admonition: "Keep thy heart with all diligence for out of it are the issues of life."

**A Shorter Surgery.** By R. J. McNeill Love, M.B., M.S., F.R.C.S. Second Edition. Pp. viii., 371. Illustrated. London: H. K. Lewis & Co. Ltd. 1930. Price 16s.—This is essentially a cram book for the senior student. The amount of information crowded into so small a space is astonishing, but the reader must have already a considerable knowledge of surgery before being able to appreciate its value. The section on ulcers and gangrene is too limited to be of any real use and might well have been omitted. In many instances the description of treatment is so brief that it fails to convey any useful information; for instance, Sayre's method of treating a fractured clavicle is the only one described, and in dealing with certain fractures of the humerus Middeldorpf's triangle is recommended without mention of the serious disadvantage of internal rotation of the distal bone. Treatment is so important a subject that it would seem best omitted altogether if space does not permit of efficient description. In spite of numerous *e.g.'s*, *i.e.'s* and etc.'s, it is a very readable book, with here and there a touch of humour, and in the right hands would be of undoubted use in facilitating revision. In our opinion interleaving with blank pages for the addition of the student's own notes would more than double the value of the book.

**Diabetes Mellitus and its Dietic Treatment.** By MAJOR B. D. BASU, I.M.S. Fifteenth Edition. Edited by L. M. BASU, M.B. Pp. vi., 160. Allahabad: Dr. L. M. Basu, M.B., Bhuvaneshwari, Allahabad. 1930. Price 2 roupees.—Major B. D. Basu, I.M.S., has been well known in India for many years for his writings on diabetes and on the history of India. The work under review was first published in 1909, and it has now reached the fifteenth edition. Major Basu has for many years insisted on the important part played by intestinal toxæmia in the production of diabetes, and he makes the interesting suggestion that the people of India, being for the most part vegetarians, require more common salt than meat eaters. But, unfortunately, they cannot afford to consume salt in sufficiently large quantities because salt is very heavily taxed. Hitherto we had regarded Gandhi's selection of the salt-tax for his crusade as being political rather than physiological in its origin. Major Basu's remark starts a new train of thought, and one begins to wonder whether the salt-tax is in reality high enough to put a sufficiency of sodium chloride beyond the purchasing power of any section of the Indian public. Major Basu has been helped by his son, Dr. L. M. Basu, in the preparation of this edition, and an admirable short account of insulin and its use has been included. This has added considerably to the value of a book which has achieved a wide popularity amongst Indian doctors.

**Percussion of the Chest.** By J. B. MACDOUGALL, M.D., F.R.C.P. Pp. viii., 143. Illustrated. London: H. K. Lewis & Co. Ltd. 1929. Price 6s.—This short account of the difficult art of percussion is written with the object of pointing out the advantages of "light percussion." The author acknowledges his debt to the pianoforte technique of Tobias Matthay, and considers that the successful exponent of percussion requires not only a "good ear" but also a working knowledge of the intricacies of touch comparable to that of a pianoforte player. He insists throughout that percussion depends upon perfection of technique for the best results. It is doubtful, indeed, whether he has not overloaded his book with acoustic problems and discussions on the four basic features of sound, namely, intensity, quality, duration and pitch. Dr. McDougall makes many references to musical instruments of various kinds, such as the human voice, the violin and the pianoforte, but he makes no reference to "tympani" or the "kettle-drums." There is no instrument in the orchestra which throws more light on the difficulties of thoracic percussion, and without knowledge of the intricacies of tuning tympani whilst the orchestra is playing, no medical author should invoke the aid of orchestral illustration to explain the art of diagnostic percussion. The book has an introductory chapter dealing with the history of percussion. The majority of doctors do not derive the same detailed information from percussion as Dr. McDougall does; a perusal of his book will perhaps explain why. To those who have a knowledge of acoustics and music the book will prove most interesting.

**The Treatment of Tuberculosis with Umckaloabo.** By ADRIEN SECHEHAYE. Pp. xvi., 162. London: B. Fraser & Co. 1930. Price 5s.—Probably many of our readers will remember the notice attracted by the libel actions launched by Mr. Stevens against the British Medical Association, based on their analysis of umckaloabo as published in *Secret Remedies*. And not a few regret that the Association considered it necessary to adopt the defence "Because analysis has failed to reveal any active principle the drug must be inert": an attitude more likely to impress a lay jury than any scientific body. The present book reprints much of the evidence tendered at the trials, as to which the reader is left to form his own opinion. The same capacity for judgment is requisite in dealing with the bulk of the book, which relates the clinical histories of a long series of cases treated with Steven's consumption cure. The author is undoubtedly enthusiastic, but tries hard to give a fair review of the effects of treatment; it is difficult, without undertaking a similar lengthy practical investigation, to criticize or contradict him.

**Medico-Legal Problems.** By LORD RIDDELL. Pp. viii., 100. London: H. K. Lewis & Co. Ltd. 1929. Price 5s.—This book contains four papers which have been published at different times in the *Transactions of the Medico-Legal Society.* The first deals with the legal responsibility of the surgeon in particular reference to operations for the sterilization of women, in the second he discusses the ethical legal and medical aspects of procuring abortions, whilst the fourth paper considers the sterilization of the unfit. The third paper is entitled the "Law and Ethics of Medical Confidences," and is much the most varied and interesting of the four. Lord Riddell's attitude to the contention that a doctor should not be forced to reveal professional confidences in the witness reveals an odd mentality. "We must recognize," he writes, "that the rules regarding them (*i.e.* medical confidences) exist for the welfare of the community and not for the aggrandisement or convenience of a particular class." It is a new idea that a doctor hesitates to divulge medical confidences for the aggrandisement or convenience of a particular class. Curiously enough, Lord Riddell recognizes that the privilege accorded to communications between solicitor and client is that of the client and not of the solicitor. If medical confidences were regarded as privileged communications of the patient it is difficult to see how the doctors would be aggrandised any more than solicitors are under the present rules. It is most interesting to read these papers, for, as Lord Riddell says, he is not speaking as a practising lawyer or a doctor, but as a member of the public.

**The Cosmetic Treatment of Skin Complaints.** By Professor E. KROMAYER. Second Edition. Pp. viii., 110. Illustrated. London: Oxford University Press. 1930. Price 6s. 6d.—This is the second edition of a small book dealing with the cosmetic side of dermatology. Professor Kromayer is the inventor of the well-known Kromayer Quartz lamp. This work does not pretend to be an exhaustive treatise on the dermatoses named, but is full of practical suggestions as to their treatment, especially by various physical means, such as skin punches, rasps, drills, and, of course, X-rays, radium and the Kromayer lamp. With the experience of thirty years to prove the advantage of the methods suggested, the author can write with authority, and his advice on many points is sound. The book serves its purpose well, as introducing alternative methods of removal of blemishes which may cause so much mental distress.

**The Morphine Habit and its Painless Treatment.** By G. LAUGHTON SCOTT, M.R.C.S. Pp. viii., 94. London: H. K. Lewis & Co. Ltd. 1930. Price 5s.—In this monograph the author explains his method of treating the morphia habit by gradual reduction of the dose, together with the administration of belladonna and hyoscine, and with large doses of luminal to ensure sleep. The dose of belladonna and hyoscine is varied according to the patient's tolerance, and every effort is made not to disturb the mental equilibrium. It is claimed that this method much reduces the discomfort and shock of withdrawal, and the average length of the cure is reduced to about twenty-eight days. It is probable that the minute attention to detail, and the constant personal supervision entailed by Dr. Scott's regime, contribute to a very large degree to the success of this method of treatment.

**Radium and Cancer (Curietherapy).** By D. C. L. FITZWILLIAMS, C.M.G., F.R.C.S. Pp. viii., 172. Illustrated. London: H. K. Lewis & Co. Ltd. 1930. Price 12s. 6d.—Books on this subject are pouring out from the press just now, all saying much the same thing, and representing much the same phase of experience. We have reviewed several in the pages of this *Journal*. Every surgeon who proposes to use radium—and who, at the present time, does not ?—should make himself acquainted with the contents of one of them, but nothing can take the place of personal experience. The volume before us is concise (169 pages), practical and adequately illustrated. It contains an interesting paragraph or two on the commercial sources and fluctuating prices of radium. We note that the writer, who is surgeon to the Mount Vernon Cancer Research Hospital, relies on block dissection rather than radium for malignant glands of the neck, and does not mention any need for lead screens to protect the jaws or palate during curietherapy of the tongue. He expects, and justly, that the knowledge that radium is available, instead of a mutilating operation, will give courage to some poor creatures, who up till now have kept the knowledge that they have a cancer of the breast to themselves for years, to come up earlier for treatment. Fitzwilliams treats the breast with radium, but clears out the axilla surgically. The glands in Hodgkin's disease rapidly disappear, but recurrence is the rule. A good account of the two schools of opinion concerning the treatment of cancer of the uterus is contributed by Dr. Malcolm Donaldson. The book suffers much from bad proof-reading. We have never reviewed a book with so many mistakes in spelling and misprints—two in consecutive lines on page 2. We read of metastases being "sewn" instead of "sown." The misuse of commas is most irritating; they are inserted copiously in the place of semi-colons, brackets and even hyphens; one even creeps in to divide asunder the honourable name of Lazarus-Barlow! Some sentences lack a verb. However, this must be judged as a text-book of surgery, not as an essay in English.

**Radium in General Practice.** By A. JAMES LARKIN, B.Sc., M.D. Pp. 304. Illustrated. New York: Paul B. Hœber. 1929. Price $6.00.—This book, belying the title, is scarcely a book of reference for the general practitioner, though it must prove of value to the enthusiastic radio-therapeutist. After all, the practitioner wants to know just what radium is offering for a disease, other than the already well-known methods of treatment. In this particular the information is partly forthcoming, though no references are made as to the relative values of radium and other therapies. Naturally, the details of the physics of the subject are given little space in such a book. At the same time a great bulk of the volume is taken up with details of the actual application of radium in different forms of disease. Even now such details are ever changing, and they cannot be of much value to the practitioner. The treatment of disease, if it is to be rational, must be based on a clear appreciation of pathology, and in this book the outlines of the pathology of each subject are clearly given. (One can only wish that radiotherapy was always based on sound pathological lines.) The reading of the book is made tiresome by the endless repetition of two sentences referring the reader to a few pages in the introduction. No particular order is observed in the volume—it is difficult to see why carcinoma of the œsophagus is included in the section on "General Diseases." There is, as is usual in American books, a very useful and extensive bibliography, and while we must recognize that the bulk of radium work has been done outside this country, it is unfortunate that the references are mainly to American workers. Both paper and print are excellent. There are hardly any diagrams, and the few there are do not add materially to what is rather an expensive book.

**The Dramatic in Surgery.** By G. GORDON TAYLOR, O.B.E., M.S. Pp. 88. Illustrated. Bristol: John Wright & Sons Ltd. 1930. Price 12s. 6d.—This is a book of absorbing interest —a record of great achievements in the face of adverse circumstances. It should find its place on the bookshelf of all practising surgeons if only to serve as a reminder that their art is not a mere routine, but a profession which may at any moment call for the highest degree of courage, judgment and skill. Mr. Gordon Taylor, looking back over thirty years of practice, and that of his surgical confrères the world over, has collected a miscellaneous series of surgical instances of the dramatic, which, apart from anecdotal interest, have a very real value to the surgeon.

**Physical Signs in Clinical Surgery.** By HAMILTON BAILEY, F.R.C.S. Second Edition. Pp. xviii., 268. Illustrated. Bristol: John Wright & Son Ltd. 1930. Price 21s.— Students who know their theoretical work fairly well are often, and rightly, "plucked" in their examinations because they do not know how to examine a patient. They will avoid this fate if they read and put into practice the contents of this excellent book. If they did no more than glance through the numerous illustrations they would learn much. We are not at all surprised that many a general practitioner has also found it very useful. A few of the clinical signs described are of dubious value of course, but it is better to know too many than too few, and any help that enables the doctor to make an accurate diagnosis by examining the patient, instead of trusting all to the skiagraphist and the pathologist, is to be welcomed.

**Nasal Catarrh.** By W. STUART LOW, F.R.C.S. Pp. x., 84. Illustrated. London: H. K. Lewis & Co. Ltd. 1930. Price 5s.—In this little book the author sets out to maintain the possibility and the desirability of curing all forms of nasal catarrh. He attempts nothing startling—indeed, one might say nothing that has not been for long the ordinary teaching of the text-books. His work is particularly notable for the stress laid on conservation of tissue and especially mucous membrane. Only bland applications are permissible or indeed useful. In common with most rhinologists he finds little advantage in vaccines. A book that the student or young graduate may find well worth reading.

**Anæsthesia and Anæsthetics.** By F. S. ROOD, M.B., B.S., and H. N. WEBBER, M.A., B.Ch. Pp. xi., 292. Illustrated. London : Cassell & Co. Ltd. 1930. Price 14s.—This book is based almost entirely upon the clinical experience of the authors and their hospital colleagues, and space has not been sacrificed to dissertations upon history or theory. In perusing its pages one's attention is early held by the attractive account of the respiratory phenomena occurring during anæsthesia, and of the various reflexes involved which may be the cause of so much embarrassment on the part of the administrator. General emergencies and complications are described with clearness that should be a great help to the student in recognizing and treating them. The clinical nature of the work is well maintained, and in the chapter entitled "A Typical Ether Case," with its picture of the events associated with induction, maintenance and recovery, though some readers will probably feel that an excessive saturation with ether has been suggested in the remark that for almost all operations abolition of the pupil light reflex is necessary. It is satisfactory to find that considerable attention has been paid to the apparatus for administering nitrous oxide, because a knowledge of the mechanism seems essential to success, but we venture to suggest that an error has crept into the illustration of Hewitt's valve in the re-breathing position. The subject of local and regional anæsthesia is clearly described with a welcome amount of technical detail, but under spinal analgesia it is noticeable that novocain is not mentioned. The book is agreeably written, well printed and adequately illustrated.

**The Extra Pharmacopœia. Vol. II.** By MARTINDALE and WESTCOTT. Nineteenth Edition. Pp. xxxviii., 759. London : H. K. Lewis & Co. Ltd. 1929. Price 22s. 6d.—Volume II. of *The Extra Pharmacopœia* is as usual a mine of information, largely chemical and analytical. The structure of the atom, foodstuffs, including advances in baking bread, vitamins, the essential oils, opotherapy, analysis organic and inorganic, the theory of drug synthesis, sterilization, antiseptic powers of disinfectants, ionization, radiology and radium therapy, balneotherapy, bacteriology and patent medicines are the titles of the larger sections of the book, and indicate its protean interest. Throughout facts, tables of facts, reports, references, analyses, supply the bulk of the matter, and produce a work of reference invaluable and unique.

**The Snake Park.** By F. W. FITZSIMONS (Director of Port Elizabeth Museum). Also **Snake Venoms : Their Therapeutic Uses and Possibilities.** By F. W. FITZSIMONS.—Probably many readers are familiar with the name of Mr. FitzSimons, the authority on the poisonous snakes of South Africa, and will welcome this account of the interesting work done at the snake park of Port Elizabeth. The prime object of the researches conducted at the park was to discover an anti-venom serum to replace the many useless "cures" sold for the treatment of snake bite ; and investigations were further extended to the possible therapeutic value of the venom itself, and owing to certain reported instances in which subjects of epilepsy, who had survived snake bite, were found subsequently to be wholly or partially free from seizures, a large number of epileptics were treated with venom and gave remarkable results. For therapeutic purposes the venom is subjected to processes of separation and blending. The author claims that protein shock has little or nothing to do with the action of the resulting product which is known as "Venene." The brochure entitled *Snake Venoms* is a reprint of a paper read by Mr. FitzSimons before the British and South African Association at Cape Town, in July, 1929.

**The Medical Annual, 1930.** Edited by CAREY F. COOMBS, F.R.C.P., and A. RENDLE SHORT, F.R.C.S. Forty-eighth Edition. Pp. c., 652, 167. Illustrated. Bristol : J. Wright & Sons Ltd. 1930. Price 20s.—We heartily welcome again this standard work. All of us feel the difficulty of keeping abreast with the many recent advances in medical knowledge, and there is no better help in this than the study of these *Annuals*, where most recent discoveries are discussed and given in abstract with references for further research. Although this work has nearly attained its fiftieth year, growth still takes place in it. It now contains over 900 pages, 700 of which are taken up by the dictionary of practical medicine. This volume, by the way, records the death of the founder of the *Annual*, Dr. Percy R. Wilde, who continued to contribute till his decease last year. A few days before his death he wrote of the 1929 volume : "I think it is quite the best." The surgical articles this year are particularly interesting, *e.g.* Leriche's account of periarterial sympathectomy and the relief of pain given by it, Hey Groves on fractures, the cure of sundry cases of congenital jaundice, the tannic acid treatment of burns and the use of diathermy in cerebral tumours. One is glad to see Hutchison criticizing the fanatics on intestinal toxæmia, and pointing out that the bowel contents are after all outside the tissues, and absorption from them is so rigidly controlled that poisons like tetanus and diphtheria in them are kept out of the tissues more perfectly than when smeared on the cracked skin of the patient's hand. We must point out the value of the lists given here of current periodicals, of the books of the year, and of new preparations and inventions, as well as the directories of sanatoria, homes and medical trades.

